# Complete Mitochondrial Genome of the Araucanian Herring, *Strangomera bentincki* , Norman, 1936 (Teleostei: Clupeiformes: Clupeidae): Phylogenetic Analysis and Implications in Fishmeal Traceability

**DOI:** 10.1002/ece3.72629

**Published:** 2025-12-08

**Authors:** Yessenia Reinoso, Cynthia M. Asorey, María Angélica Larraín, Cristian Araneda

**Affiliations:** ^1^ Food Quality Research Center Universidad de Chile Santiago Chile; ^2^ Doctorado en Nutrición y Alimentos, Facultad de Ciencias Químicas y Farmacéuticas Universidad de Chile Santiago Chile; ^3^ Departamento de Ciencia de los Alimentos y Tecnología Química, Facultad de Ciencias Químicas y Farmacéuticas Universidad de Chile Santiago Chile; ^4^ Center for Ecology and Sustainable Management of Oceanic Islands (ESMOI) and Sala de Colecciones Biológicas (SCBUCN) Universidad Católica del Norte Coquimbo Chile; ^5^ Departamento de Producción Animal, Facultad de Ciencias Agronómicas Universidad de Chile Santiago Chile

**Keywords:** Araucanian herring, Clupeoid, common sardine, fisheries, vertebrata

## Abstract

Araucanian herring or common sardine, *S. bentincki* (Norman 1936), is a pelagic fish whose main processed products are fishmeal and oil. The complete mitogenome was obtained from one specimen collected at Isla Mocha, Chile (−38.497, −73.619). The size of the mitochondrial genome is 16,758 bp and consists of 37 genes typical in fish: 13 coding sequences (CDS), two ribosomal RNA (rRNA) genes, 22 transfer RNA (tRNA) genes, and a non‐coding control region. The gene organization and arrangement are similar to those of other fish species in the Clupeidae and other Teleostei families. Phylogenetic analysis inferred by the maximum likelihood method using the complete mitochondrial genome of 26 species related to *Strangomera bentincki* generated a strongly supported clade with *Sprattus muelleri* and *Sprattus antipodum* . Comparative analysis of the mitogenome reveals genetic divergences of 7.8% with *S. muelleri* and 7.7% with *S. antipodum* , which are the closest taxa, indicating that they are likely sister species. Our results provide a mitochondrial genome sequence to support the development of DNA‐based traceability tools for processed foods containing Araucanian herring. This mitogenome is a valuable genetic resource for future phylogenetic studies involving Clupeoid species.

## Graphical abstract

Araucanian herring ( *Strangomera bentincki* ) is a pelagic fish whose main processed products are fishmeal and oil. The complete mitogenome is 16,758 bp, consisting of 13 coding genes, two rRNAs, 22 tRNAs, and the control region. Phylogenetic analysis supported a monophyletic clade with *Sprattus muelleri* and *Sprattus antipodum* . Our result provides information for developing DNA‐based traceability tools to control quota landings in fishmeal production, avoiding overexploitation.

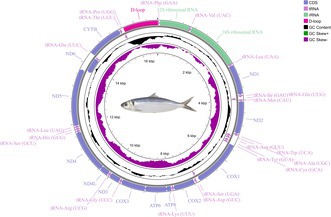

## 1 Introduction

Clupeoid fishes (sardines, herring, sprats, shads) include more than 300 species grouped in 80 genera and 10 families (*Engraulidae*, *Dorosomatidae*, *Alosidae*, *Pristigasteridae*, *Ehiravidae*, *Clupeidae*, *Dussumieriidae*, *Spratelloididae*, *Chirocentridae*, and *Denticipitidae*) (FAO 2019). They inhabit temperate, tropical, and cold marine waters worldwide (Whitehead 1985). The Clupeidae family comprises eight accepted genera (*Clupea*, *Ethmidium*, *Hyperlophus*, *Potamalosa*, *Ramnogaster*, *Sprattus*, and *Strangomera*), with 16 valid species (WoRMS 2025). Within the Clupeidae family, nine marine species ( *Clupea harengus* , *Clupea pallasi* , *Ethmidium maculatum* , *Hyperlophus vittatus* , *Ramnogaster arcuata* , *Sprattus antipodum* , *Sprattus fuegensis* , *Sprattus sprattus* , *Strangomera bentincki* ) are of interest to fisheries. They are included in the FAO's global capture and aquaculture production statistics list (FAO 2019).

The Araucanian herring or common sardine, *S. bentincki* (Norman 1936), is a pelagic fish geographically distributed in coastal waters of Chile (Coquimbo to Talcahuano) in the FAO fishing zone N° 87 (FishBase 2017). This cold‐water species is exclusively fished in Chile. Based on the available indicators and trends for 2024, the *S. bentincki* fishery in the area between the Valparaíso (33° S) and Los Lagos (44° S) regions is classified as overexploited (SUBPESCA 2025). It is a rich source of high‐quality proteins with high biological value and omega‐3 fatty acids, trace elements, and fat‐soluble vitamins (Garcia et al. 2019; Singer et al. 2016). In Chile, 99.91% of *S. bentincki* is used as a raw material for fishmeal production, and to a lesser extent, for direct human consumption (0.02% as chilled and refrigerated, and 0.07% as frozen) (SERNAPESCA 2023). Between 2018 and 2023, landings of this resource averaged 318 thousand Tonnes, ranging from ~223 (2022) to ~410 (2023) (FAO 2025). The catch is regulated by annual fishing quotas (SUBPESCA 2024). As the primary use of this species is in fishmeal production, commonly blended with *Engraulis ringens* , *Scomber japonicus* , and *Trachurus murphyi* (SERNAPESCA 2024), it is important to have analytical methods to identify the species and explore the possibility of quantifying their proportion in fishmeal, thereby inferring the biomass per species and ensuring compliance with the law.

DNA analysis is a reliable molecular approach for species authentication and regulatory enforcement in fisheries and aquaculture (Martinsohn et al. 2019). In seafood, mitochondrial *cytochrome C oxidase subunit 1* (*cox*1), *cytochrome b* (*cytb*), *16S rDNA*, and *12S rDNA* genes are the most widely used DNA barcoding targets (Cawthorn et al. 2012; Tinacci et al. 2018). However, without the complete mitochondrial genome, other barcode genes cannot be tested for species identification. Three out of 10 species of Clupeidae, considered commercially valuable (FAO 2019), do not have their mitochondrial genome available in online databases: *S. fuegensis* , *R. arcuata* , and *S. bentincki* . This information gap hinders the development of new analytical methods based on barcoding or mini‐barcoding to enforce fishing quotas and to prevent fraudulent practices in processed foods, such as fishmeal. In this work, we sequenced and annotated the complete mitochondrial genome of *S. bentincki* . Additionally, this genome was compared with other species from the family Clupeidae and species used in fishmeal production in Chile. Although this study does not include proof‐of‐concept for traceability applications, it provides a crucial genomic resource to fill an important gap in public databases, enabling the design of reliable DNA‐based assays for species authentication in fishmeal. Access to full mitogenomes of both target and non‐target taxa will allow the identification of diagnostic regions with adequate interspecific variability, thereby avoiding false positives in traceability systems (Cawthorn et al. 2012; Diver et al. 2024).

## 2 Materials and Methods

### 2.1 Sampling

**FIGURE 1:**
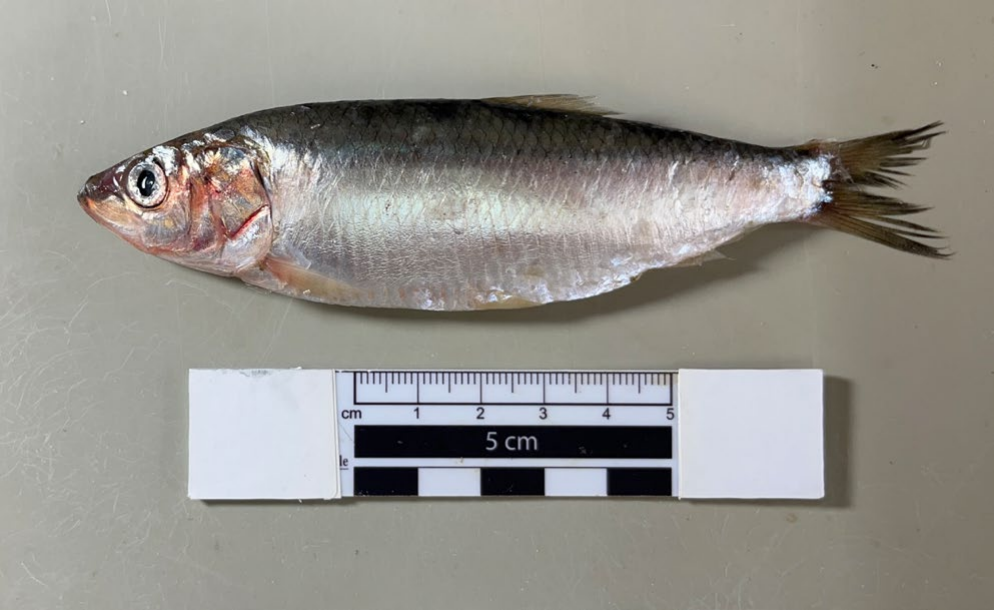
Araucanian herring, *Strangomera bentincki* used for the mitochondrial genome sequencing (photo credit: Cristian Araneda).

One specimen of the common sardine, *S. bentincki* (Norman 1936), was collected at Isla Mocha, Chile (−38.497, −73.619) in February 2024 (Figure 1). It was identified morphologically, as described by the Instituto de Fomento Pesquero, Chile (IFOP 2020), and preserved in absolute ethanol and stored at −20°C until analysis. This specimen was deposited in the ichthyological collection of the Chilean National Museum of Natural History under the voucher number MNHNCL ICT 8212.

### 2.2 DNA Extraction, Sequencing, and Assembling

Genomic DNA (gDNA) was extracted using the phenol‐chloroform method from a finclip (Taggart et al. 1992), and its purity and concentration were assessed using a NanoDrop 2000 Spectrophotometer (ThermoFisher) and a Qubit fluorometer (ThermoFisher), respectively. The library was prepared using gDNA with the Illumina DNA Prep Kit (Illumina Inc.) according to the manufacturer's protocol. Paired‐end sequencing (150 × 2 cycles) was performed using the iSeq100 System (Illumina Inc.), available at the Laboratory of Genetics and Biotechnology in Aquaculture (Department of Animal Production, Faculty of Agronomic Sciences, University of Chile). Sequenced reads were trimmed using the BBDuk plugin v.38.84, and a de novo assembly was performed with Geneious Prime 2025.0.3 assembler without reference genome (https://www.geneious.com), to obtain the mitogenome consensus contig. The overlapping and ends were manually edited to obtain the circularized mitogenome. Finally, trimmed reads were mapped again to check the assembly and generate the final version of the mitochondrial genome. If well, there are other specific assemblers for mitochondrial genomes, such as GetOrganelle (Jin et al. 2020), which have good performance (Ni et al. 2025), we prefer to use Geneious Prime Assembler because it allows us to obtain other contigs containing barcode genes, such as the ribosomal cistron. An alignment comparison between the assemblies obtained with Geneious Prime and GetOrganelle is shown in Figure S1.

The final contig containing the mitochondrial genome was manually annotated by aligning it with the complete mitochondrial genome of *S. muelleri* (NC_016669). The annotations were confirmed with other complete mitochondrial reference genomes belonging to small pelagic species ( *S. muelleri* [NC_016669], *S. sprattus* [NC_009593], *S. antipodum* [NC_016673], *S. japonicus* [NC_013723], *Sardinops sagax* [NC_057117], *E. maculatum* [NC_016710], *T. murphyi* [PP533446], *Trachurus symmetricus* [OR482443], *E. ringens* [NC_042728]). These annotations were compared with those obtained from MITOS2 (Bernt et al. 2013; Donath et al. 2019), which was run on the Galaxy platform (The Galaxy Community 2024), and with MiFish v2025.06 (Iwasaki et al. 2013; Zhu et al. 2023).

The circular mitochondrial genome map of *S. bentincki* was generated using the GCView program (https://stothardresearch.ca/cgview/) (Stothard and Wishart 2005). The secondary tRNA structures were drawn with tRNAscan‐SE 2.0 online (https://lowelab.ucsc.edu/tRNAscan‐SE/) (Lowe and Chan 2016) using the configuration for a vertebrate mitochondrial (sequence source), default search mode, query sequence in FASTA format.

### 2.3 Relative Synonymous Codon Usage Analysis

The Codon Usage tool within the Sequence Manipulation Suite web server was used to determine the codon usage for all 13 protein‐coding genes in the mitochondrial genome of *S. bentincki* (https://www.bioinformatics.org/sms2/codon_usage.html) (Stothard 2004). The EZcodon tool within EZmito was used to determine the relative synonymous codon usage within the 13 coding genes (http://ezmito.unisi.it/ezcodon) (Cucini et al. 2021; Lee 2018).

### 2.4 Phylogenetic Analysis

The phylogenetic analysis considered the complete mitochondrial genome of 23 Clupeiformes species, along with one Carangiformes species and two Scombriformes species as outgroups. Several criteria were considered when selecting species from the aforementioned orders. First, a maximum of three marine species were chosen from each of the 10 taxonomic families of Clupeiformes, according to the Statistical Classification of Aquatic Animals and Plants for Fisheries Purposes (FAO 2019): *Engraulidae*, *Dorosomatidae*, *Alosidae*, *Pristigasteridae*, *Ehiravidae*, *Clupeidae*, *Dussumieriidae*, *Spratelloididae*, *Chirocentridae*, and *Denticipitidae*. In addition, the seven most commonly caught pelagic species listed in the report “The State of World Fisheries and Aquaculture 2024” (FAO 2024) are included (Table 1).

**TABLE 1:** Species, genus, order, NCBI access number, and reference of the species included in the phylogenetic analyses.

Species	Family	Order	NCBI_access number	References
*Strangomera bentincki*	Clupeidae	Clupeiformes	PV863448	This work
*Clupea harengus*			NC_009577.1	Lavoué et al. (2007)
*Clupea pallasi*			NC_009578.1	Lavoué et al. (2007)
*Sprattus sprattus*			NC_009593.1	Lavoué et al. (2007)
*Sprattus antipodum*			NC_016673	Lavoué et al. (unpublished)
*Sprattus muelleri*			NC_016669	Lavoué et al. (unpublished)
*Ethmidium maculatum*			NC_016710	Lavoué et al. (unpublished)
*Hyperlophus vittatus*			NC_016671.1	Lavoué et al. (unpublished)
*Sardinella fijiensis*	Dorosomatidae		NC_044472	Wang et al. (2019)
*Sardinella jussieu*			KY964441	Sektiana et al. (2017)
*Sardinella gibbosa*			NC_037131	Sebastian et al. (2018)
*Sardinops sagax*	Alosidae		NC_057117	Tang and Chen (2021)
*Sardina pilchardus*			NC_009592	Lavoué et al. (2007)
*Brevoortia tyrannus*			NC_014266.1	Lavoué et al. (2010)
*Engraulis ringens*	Engraulidae		NC_042728	Sun (2019)
*Coilia mystus*			NC_019644.1	Bo et al. 2013
*Thryssa vitrirostris*			NC_039819.1	Du et al. (2019)
*Ehirava fluviatilis*	Ehiravidae		NC_016717.1	Lavoué et al., (unpublished)
*Etrumeus sadina*	Dussumieriidae		NC_009583.1	Lavoué et al. (2007)
*Dussumieria elopsoides*			NC_035063	Lavoué et al. (2017)
*Spratelloides delicatulus*	Spratelloididae		NC_009588.1	Lavoué et al. (2007)
*Spratelloides gracilis*			NC_009589.1	Lavoué et al. (2007)
*Jenkinsia lamprotaenia*			NC_006917.1	Ishiguro et al. (2005)
*Trachurus murphyi*	Carangidae	Carangiformes	PP533446	Asorey et al. (2024)
*Scomber japonicus*	Scombridae	Scombriformes	NC_013723	Catanese et al. (2010)
*Scomber scombrus*			NC_006398.1	Takashima et al. (2006)

The maximum‐likelihood phylogenetic reconstruction was obtained using the plugin PhyML 3.3.2 with the following settings: substitution model = GTR, bootstrap = 1000, the proportion of invariable sites = estimated, gamma distribution parameter = estimate, optimize = Topology/length/rate (Guindon et al. 2010).

## 3 Results

### 3.1 Sequencing

A total of 6,110,604 paired reads (Q20 = 99.9%, Q30 = 97.1%, 2 × 150 bp) were obtained from one iSeq 100 flow cell. The complete mitochondrial genome of *S. bentincki* was assembled with 5289 reads (mean coverage = 36.9, min = 1, max = 74) (Figure S2), yielding a circular sequence of 16,758 bp (GenBank accession no. PV863448).

### 3.2 Mitochondrial Genome

**FIGURE 2:**
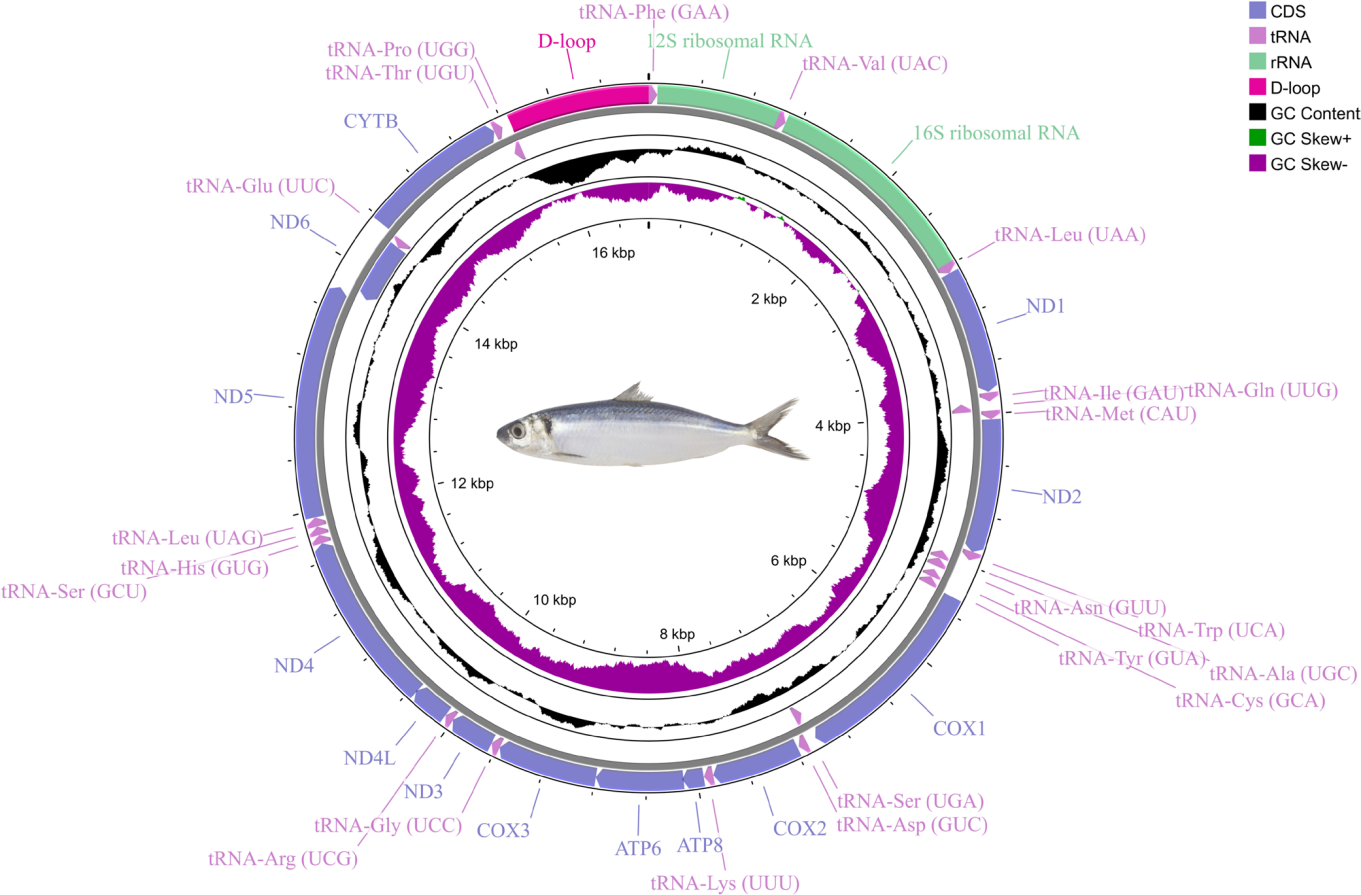
Circular map of the *Strangomera bentincki* mitogenome. The H strand genes are drawn toward the outside of the chromosome circle, and the L strand genes are drawn toward the inside.

The complete mitochondrial genome of *S. bentincki* was a typical circular molecule of 16,758 bp in size. Both the order and arrangement of the mitogenome genes were identical to those of the other five species of Clupeidae used in this study ( *S. sprattus* , *S. muelleri* , *S. antipodum* , *C. pallasii* , and *C. harengus* ). It consisted of 13 protein‐coding genes, two ribosomal RNA units, 22 transfer RNA genes, and one non‐coding region (control region) in the following order: *tRNA‐Phe*, *12 rRNA*, *tRNA‐Val*, *16S rRNA*, *tRNA‐Leu* (*UAA*), *ND1*, *tRNA‐Ile*, *tRNA‐Gln*, *tRNA‐Met*, *ND2*, *tRNA‐Trp*, *tRNA‐Ala*, *tRNA‐Asn*, *tRNA‐Cys*, *tRNA‐Tyr*, *COX1*, *tRNA‐Ser* (*UGA*), *tRNA‐Asp*, *COX2*, *tRNA‐Lys*, *ATP8*, *ATP6*, *COX3*, *tRNA‐Gly*, *ND3*, *tRNA‐Arg*, *ND4L*, *ND4*, *tRNA‐His*, *tRNA‐Ser* (*GCU*), *tRNA‐Leu* (*UAG*), *ND5*, *ND6*, *tRNA‐Glu*, *CYTB*, *tRNA‐Thr*, *tRNA‐Pro*, *D‐loop* (control region) (Figure 2).

The base composition of the L strand (light or minor) is as follows: A, 25.8%; C, 28.6%; G, 20.1%, and T, 25.5%. The *16S rRNA* has a length of 1686 bp and is located between *tRNA‐Phe* and *tRNA‐Val*, while the *12S rRNA* gene shows a size of 954 bp and is located between *tRNA‐Val* and *tRNA‐Leu* (*UAA*). The protein‐coding genes (*ND1*, *ND2*, *COX2*, *ATP8*, *ATP6*, *COX3*, *ND3*, *ND4L*, *ND4*, *ND5*, *ND6*, and *CYTB*) have a conventional start codon: ATG, while the protein‐coding gene *COX1* has GTG as a starting codon, respectively. The stop codons of the protein‐coding genes are the typical TAA codon for *ND1*, *COX1*, *ATP8*, *ATP6*, *COX3*, *ND4L*, *ND5*, and *ND6*; TAG codon for *ND2* and *ND3*; and *COX2*, *ND4*, and *CYTB* coding genes show an incomplete stop codon, T‐‐. The annotation of the *ATP6*, *COX3*, *ND3*, and *ND2* genes showed a discrepancy when using MitoFish. The stop codons for the *ATP6* and *COX3* genes were TA‐, and for the *ND2* and *ND3* genes, they were T‐ (Table S1). The 22 tRNA genes exhibit lengths ranging from 66 bp (*tRNA‐Cys*) to 75 bp (*tRNA‐Lys*), totaling 1555 bp in length (Table 2). Fourteen tRNA genes are located on the H strand: *tRNA‐Phe*, *tRNA‐Val*, *tRNA‐Leu* (*UAA*), *tRNA‐Ile*, *tRNA‐Met*, *tRNA‐Trp*, *tRNA‐Asp*, *tRNA‐Lys*, *tRNA‐Gly*, *tRNA‐Arg*, *tRNA‐His*, *tRNA‐Ser* (*UGA*), *tRNA‐Leu* (*UAG*), and *tRNA‐Thr*, while the remaining eight genes: *tRNA‐Gln*, *tRNA‐Ala*, *tRNA‐Asn*, *tRNA‐Cys*, *tRNA‐Tyr*, *tRNA‐Ser* (*GCU*), *tRNA‐Glu*, and *tRNA‐Pro* are embedded in the L strand. All tRNA genes show a clover secondary structure except *tRNA‐Ser* (*GCU*) (Figure S3), which lacks the D‐arm of the dihydrouridine stem (DHU).

**TABLE 2:** The complete mitochondrial genome of *Strangomera bentincki* with annotation.

Gene	Direction	Location		Size (bp)	Anticodon	Start codon	Stop codon
		Start	Finish				
*tRNA‐Phe*	+	1	69	69	GAA		
*12 rRNA*	+	70	1023	954			
*tRNA‐Val*	+	1024	1094	71	UAC		
*16S rRNA*	+	1095	2780	1686			
*tRNA‐Leu*	+	2781	2855	75	UAA		
*ND1*	+	2856	3830	975		ATG	TAA
*tRNA‐Ile*	+	3836	3907	72	GAU		
*tRNA‐Gln*	−	3907	3977	71	UUG		
*tRNA‐Met*	+	3977	4045	69	CAU		
*ND2*	+	4046	5092	1047		ATG	TAG
*tRNA‐Trp*	+	5091	5162	72	UCA		
*tRNA‐Ala*	−	5164	5232	69	UGC		
*tRNA‐Asn*	−	5234	5306	73	GUU		
*tRNA‐Cys*	−	5337	5.402	66	GCA		
*tRNA‐Tyr*	−	5404	5474	71	GUA		
*COX1*	+	5476	7026	1551		GTG	TAA
*tRNA‐Ser*	−	7027	7097	71	UGA		
*tRNA‐Asp*	+	7102	7171	70	GUC		
*COX2*	+	7184	7874	691		ATG	T‐‐
*tRNA‐Lys*	+	7875	7949	75	UUU		
*ATP8*	+	7951	8118	168		ATG	TAA
*ATP6*	+	8109	8792	684		ATG	TAA
*COX3*	+	8792	9577	786		ATG	TAA
*tRNA‐Gly*	+	9577	9648	72	UCC		
*ND3*	+	9646	9999	351		ATG	TAG
*tRNA‐Arg*	+	9998	10,067	70	UCG		
*ND4L*	+	10,068	10,364	297		ATG	TAA
*ND4*	+	10,358	11,738	1381		ATG	T‐‐
*tRNA‐His*	+	11,739	11,807	69	GUG		
*tRNA‐Ser*	+	11,808	11,874	68	GCU		
*tRNA‐Leu*	+	11,875	11,946	72	UAG		
*ND5*	+	11,947	13,782	1836		ATG	TAA
*ND6*	−	13,779	14,300	522		ATG	TAA
*tRNA‐Glu*	−	14,301	14,369	69	UUC		
*CYTB*	+	14,374	15,514	1141		ATG	T‐‐
*tRNA‐Thr*	+	15,515	15,585	71	UGU		
*tRNA‐Pro*	−	15,585	15,654	70	UGG		
*D‐loop*	+	15,671	16,758	1088			

*Note:* Direction: (+) Forward, (−) Reverse.

Also, an overlap between genes coding for adjacent proteins was observed in the mitochondrial genome of *S. bentincki* ; for example, this is the case of *ATP8*‐*ATP6*‐*COX3* and *ND4L*‐*ND4*, which have nucleotide overlaps of 10–1 nucleotides (ATGATACTAA‐A) and seven nucleotides (ATGCTAA), respectively. It occurs in the same chain. Another case is that of *ND5*‐*ND6*, where the observed four‐nucleotide overlap (TTAA) appears on the opposite strand.

### 3.3 Relative Synonymous Codon Usage Analysis

**FIGURE 3:**
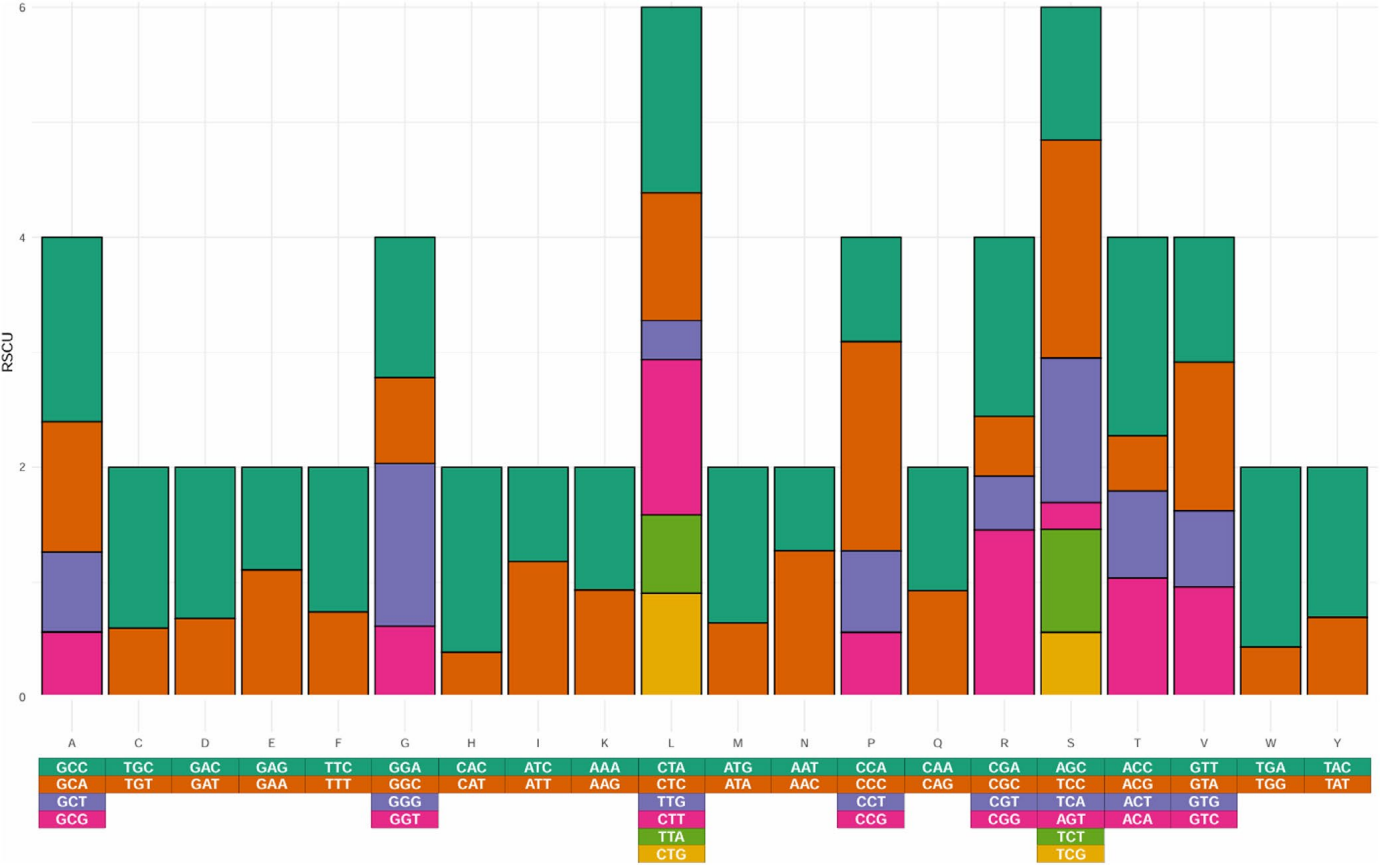
Relative synonymous codon usage (RSCU) within the protein‐coding genes of *Strangomera bentincki* .

Within the mitochondrial protein‐coding genes of *S. bentincki* , the most used codons were CTA (Leu, *n* = 166, 16.2% aa total), GCC (Ala, *n* = 150, 9.8% aa total), and TTC (Phe, *n* = 149, 6.2% aa total). The least used codons were TAA (Stop, *n* = 7, 0.2% aa total), TGT (Cys, *n* = 9, 0.8% aa total), CGT (Arg, *n* = 9, 2% aa total), AGT (Ser, *n* = 9, 6.1% aa total) (Table S2). The relative synonymous codon usage (RSCU) analysis showed that the highest RSCU values were for CCC (Pro, 1.818), ACC (Thr, 1.727), CTA (Leu, 1.614), and GCC (Ala, 1.604). The lowest RSCU values were for CCA (Pro, 0.909), CAA (Gln, 1.072), and GTT (Val, 1.086) (Figure 3).

### 3.4 Phylogenetic Relationships

**FIGURE 4:**
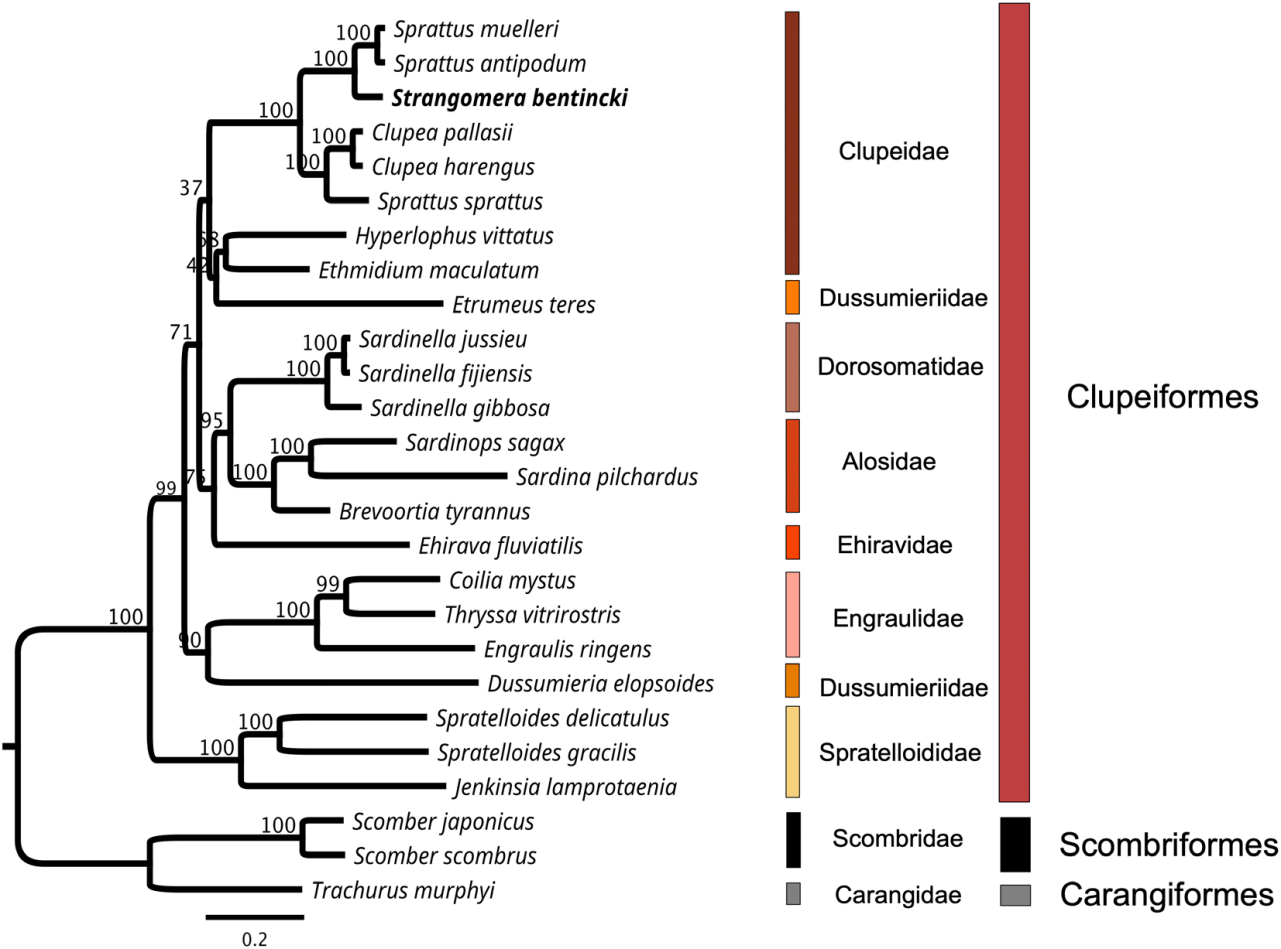
Phylogenetic tree inferred by Maximum‐likelihood phylogenetic reconstruction based on the complete mitochondrial genome (substitution model = GTR, bootstrap = 1000, proportion of invariable sites = estimated, gamma distribution parameter = estimate, optimize = Topology/length/rate).

The phylogenetic analysis was performed using 26 species, distributed across three orders and nine families. Only bootstraps (BS) over 90% are shown in the tree (Figure 4). The analyzed species, belonging to Clupeiformes, formed a well‐defined clade (BS = 100%), supporting their taxonomic status.

There is a shared evolutionary relationship among members of the Clupeidae family. It diversifies into two clades: the first consists of the *Sprattus*, *Clupea*, and *Strangomera* genera (BS = 100%), while the second is divided into two genera: *Ethmidium* and *Hyperlophus*. *S. bentincki* forms a well‐defined monophyletic group (BS = 100%) with *S. antipodum* and *S. muelleri* and is considered phylogenetically close species. Their complete mitogenomes differ by 7.7% from those of *S. antipodum* and 7.8% from those of *S. muelleri* , whereas differences with other species range from 14.6% to 29.8%.

## 4 Discussion and Conclusion

The mitochondrial genome in Teleostei fishes spans between 16 and 17 kilobase pairs (Kb) and comprises 13 conserved coding genes, two ribosomal RNAs, 22 transfer RNAs, and two non‐coding regions. In this work, we report on the complete mitogenome of *S. bentincki* , which presents an arrangement, number, and gene order similar to those described in the mitochondrial genomes of most Teleostei fishes (Bo et al. 2013; Satoh et al. 2016). We found that the start codon is ATG for most protein‐coding genes, while TAA is the predominant complete stop codon. Other stop codons found were TAG and the incomplete codon (T‐‐). The genes showing the incomplete stop codons were *COX2*, *ND4*, and *CYTB*. These results are consistent with those of Satoh et al. (2016), who noted that ATG is the predominant start codon in 250 fish species, including two species ( *Engraulis japonicus* and *Sardinops melanostictus* ) belonging to the Clupeiformes order. The same author also described seven stop codons, including those found in this work and mentioned above (Satoh et al. 2016).

The codon usage pattern in *S. bentincki* closely follows the A + T bias typical of Clupeoid mitochondrial genomes (Sebastian et al. 2022). The predominance of codons ending in A or T (e.g., CTA for Leu) suggests that mutational pressure is the primary driver of codon usage. The relatively high RSCU values for GCC (Ala) and ACC (Thr) indicate a preference for GC‐ending codons, which may reflect translational optimization or specific metabolic adaptations of *S. bentincki* to the cold water and variations in oxygen and temperature of the southeastern Pacific. Overall, these results support the hypothesis that both mutational bias and selective constraints contribute to shaping codon usage in the mitochondrial genome of Clupeid fish (Sebastian et al. 2022).

The length of the mitogenome and protein‐coding genes is within the most frequent ranges found in fish (Satoh et al. 2016). When comparing the length of the mitogenomes of the 26 species used in this study, some differences are found in the following genes: *ND6* (522–600 bp), *ND5* (1836–1839), *ND4* (1381–1382), *ND3* (349–351), *ATP6* (681–685), *ATP8* (165–168), *COX1* (1545–1563), *ND2* (1044–1047), *ND1* (974–975), *16S rRNA* (1667–1719), and *12S rRNA* (944–962). The length of the overlap nucleotides (up to 10 bp) is detected between protein‐coding genes, which coincides with that reported in other studies of mitogenomes in sardines (Sektiana et al. 2017).

Regarding the secondary structure of the 22 tRNA genes, we found that all of them have a clover shape except for *tRNA‐Ser* (*GCU*), as reported in other sardines (Lavoué et al. 2007). Mitochondrial serine *tRNA‐Ser* (*GCU*) naturally lacks a D‐arm. However, compensatory structures in the TψC arm have evolved, allowing *tRNA‐Ser* (*GCU*) to function correctly (Watanabe 2010). These compensatory structures, such as post‐transcriptional base modifications, enhance structural rigidity and non‐canonical pairings within the anticodon arm, preserving the functional geometry of the tRNA (Krahn et al. 2020; Watanabe et al. 2014).

The phylogenetic analysis using the complete mitochondrial genome presented here is consistent with other studies that used a partial fragment of *COX* *1* (Canales‐Aguirre et al. 2021) or complete mitogenomes (Lavoué et al. 2007). *S. bentincki* is the only currently recognized species within the *Strangomera* genus and forms a distinct clade with *S. muelleri* and *S. antipodum* , confirming that *Strangomera* is closely related to some southern‐hemisphere‐distributed species of *Sprattus* (Canales‐Aguirre et al. 2021). *S. bentincki* showed a 7.7% difference with *S. antipodum* and a 7.8% difference with *S. muelleri* . These differences were greater than those recorded in the family for species within the same genus (e.g., 1.8% between *S. antipodum and S. muelleri*).

In the fishmeal industry, species substitution can occur accidentally due to mixed‐species fisheries or deliberately to avoid bans and fishing quotas per species. Specifically, in Chile, the most common species used as raw material for fishmeal production are *T. murphyi* (Chilean Jack Mackerel), *E. ringens* (Peruvian Anchovy), *S. japonicus* (Chub Mackerel), and *S. bentincki* (Araucanian herring) (SERNAPESCA 2024). These species are subject to a fishing quota under Chilean fishery regulations. Consequently, accurate species identification in fishmeal is necessary for stock management and law enforcement. In this work, we report the entire mitochondrial genome of *S. bentincki* , obtained from a single specimen. Although it does not capture the intraspecific variability of the species, it is an essential reference. The *S. bentincki* mitogenome, together with those from *E. ringens* (Sun 2019), *S. japonicus* (Catanese et al. 2010), and *T. murphyi* (Asorey et al. 2024), will allow the development of new mini‐barcode markers to identify these species in fishmeal produced in Chile.

## Author Contributions

**Yessenia Reinoso:** formal analysis (equal), methodology (equal), visualization (equal), writing – original draft (lead), writing – review and editing (equal). **Cynthia M. Asorey:** formal analysis (equal), software (equal), supervision (equal), validation (equal), writing – review and editing (equal). **María Angélica Larraín:** conceptualization (equal), funding acquisition (equal), project administration (equal), supervision (equal), writing – review and editing (equal). **Cristian Araneda:** conceptualization (equal), funding acquisition (equal), methodology (equal), project administration (equal), supervision (equal), writing – review and editing (equal).

## Funding

This work was supported by the Chilean National Agency of Research and Development (ANID) through grant ANID FONDECYT Regular 1231920.

## Ethics Statement

All research followed protocols approved under ID 23697‐CQyF‐UCH by the Institutional Animal Care and Use Committee, University of Chile (“Comité Institucional de Cuidado y Uso de Animales [CICUA])”. This project was performed in adherence to ARRIVE guidelines (https://arrive guidelines.org/arrive‐guidelines).

## Conflicts of Interest

The authors declare no conflicts of interest.

## Supporting information


**Figure S1:** Alignment of *Strangomera bentincki* assemblies obtained with Geneious Prime Assembler and GetOrganelle.

**Figure S2:** Read coverage plot of *Strangomera bentincki* mitochondrial genome.

**Figure S3:** Secondary structures of the transfer RNAs in *Strangomera bentincki* .

**Table S1:** Comparison of annotation among MITOS2, MitoFish, and manual annotation.

**Table S2:** Codon Usage and RSCU analysis within the protein‐coding genes of *Strangomera bentincki* .

## Data Availability

The mitochondrial genome sequence data supporting this study's findings are available on GenBank of NCBI at https://www.ncbi.nlm.nih.gov under accession number PV863448. Row reads are available in the NCBI SRA repository under the BioProject ID PRJNA1347135 and BioSample accession number SAMN52851944.
