# Supplementary material for: Complete Mitochondrial Genome of the Araucanian Herring, Strangomera bentincki , Norman, 1936 (Teleostei: Clupeiformes: Clupeidae): Phylogenetic Analysis and Implications in Fishmeal Traceability
Source: Ecol Evol. 2025 Dec 8;15(12):e72629. doi: 10.1002/ece3.72629 (PMC12683357; doi:10.1002/ece3.72629)
Supplement: Supplementary file 2 — Figure S2: Read coverage plot of Strangomera bentincki mitochondrial genome. [file ECE3-15-e72629-s001.docx]

Complete Mitochondrial Genome of the Araucanian herring, *Strangomera bentincki*, Norman, 1936 (Teleostei: Clupeiformes: Clupeidae): Phylogenetic analysis and implications in fishmeal traceability

Yessenia Reinoso^a,b,c^, Cynthia M. Asorey^d^, María Angélica Larraín^a,c^, and Cristian Araneda^a,e^

^a^Food Quality Research Center, Universidad de Chile, Santiago, Chile.

^b^Doctorado en Nutrición y Alimentos. Facultad de Ciencias Químicas y Farmacéuticas, Universidad de Chile.

^c^Departamento de Ciencia de los Alimentos y Tecnología Química. Facultad de Ciencias Químicas y Farmacéuticas, Universidad de Chile. Independencia 8380494, Santiago, Chile.

^d^ Center for Ecology and Sustainable Management of Oceanic Islands (ESMOI) and Sala de Colecciones Biológicas (SCBUCN), Universidad Católica del Norte, Coquimbo, Chile.

^e^Departamento de Producción Animal. Facultad de Ciencias Agronómicas, Universidad de Chile. Santa Rosa 11315, Santiago, Chile.

* Corresponding author:

Cristian Araneda, e-mail: [craraned@uchile.cl](mailto:craraned@uchile.cl)

**SUPPLEMENTARY MATERIAL**


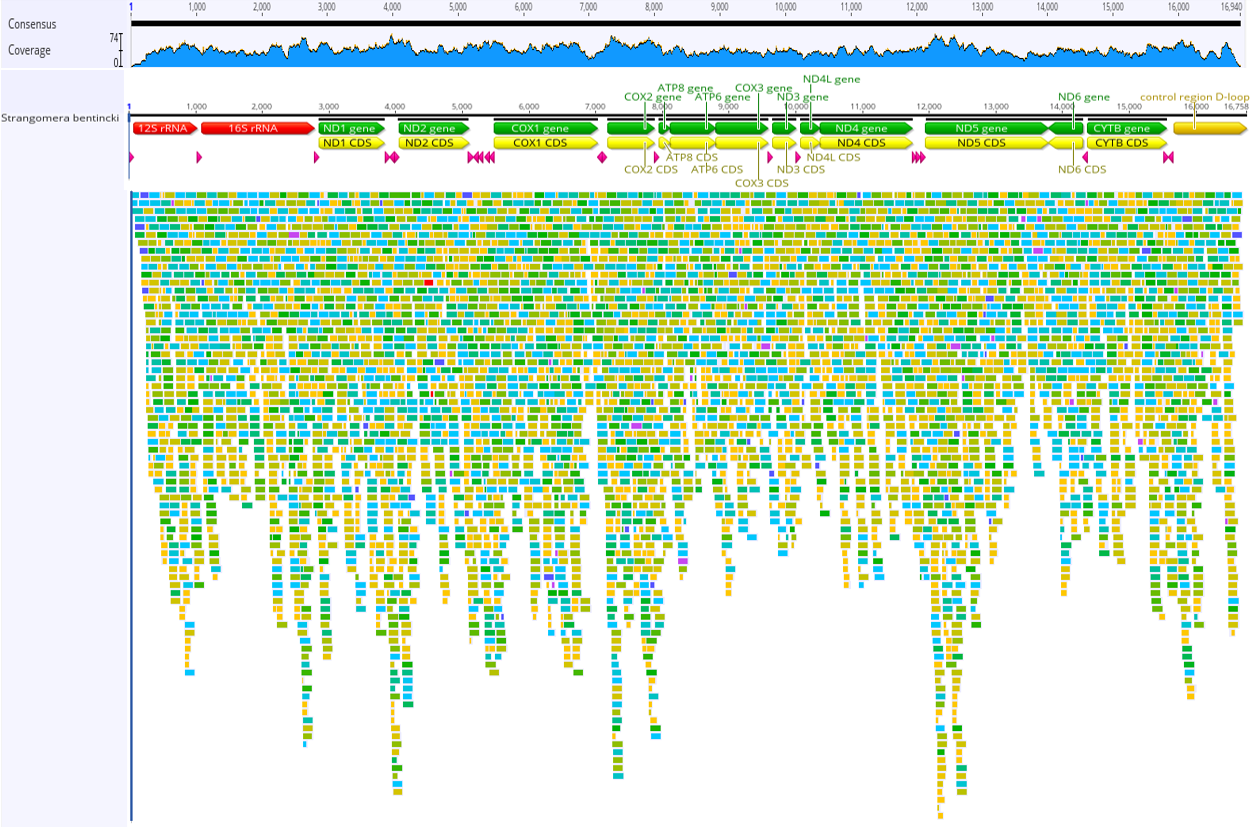


**Figure S2.** Read coverage plot of *Strangomera bentincki* mitochondrial genome
